# Blood lipids and adipokines concentrations during a 6-month nutritional and physical activity intervention for metabolic syndrome treatment

**DOI:** 10.1186/1476-511X-9-148

**Published:** 2010-12-31

**Authors:** Frédéric Dutheil, Bruno Lesourd, Daniel Courteix, Robert Chapier, Eric Doré, Gérard Lac

**Affiliations:** 1 Clermont University Blaise Pascal, BAPS, EA 3533, BP 10448, F 63000 Clermont-Ferrand, France; 2 Geriatrics department, Faculty of medicine, F 63000 Clermont-Ferrand, France; 3 Occupational medicine, Faculty of medicine, F 63000 Clermont-Ferrand, France; 4 General practitioner, thermal baths, F 63140 Châtel-Guyon, France

## Abstract

**Background:**

To report changes in body weight, total and central fat mass, metabolic, hormonal and inflammatory parameters in overweight people who participated in a six months weight loss intervention associating diet management and exercise.

**Subjects and Methods:**

Fourteen subjects (10 M, 4 F, mean age 62.9 ± 6.9 years, BMI 30.4+/- 3.8 kg/m^2^) presenting the characteristics of the Metabolic Syndrome (MS) were included in the survey. They followed a three weeks (D0 to D20) cure in a medical establishment and a six months (D20 to M3 and M6) follow up at home. During the cure, they receive a balanced diet corresponding to 500 Kcal deficit vs their dayly energy expenditure (DEE) and they exercised 2 to 3 hours per day.

At D0, D20, M3 and M6, body composition (lean mass, total and central fat mass) was analyzed with DEXA, blood pressure was taken and blood was collected to evaluate glycaemia, triglycerides, total, LDL and HDL cholesterol, insulin, leptin and adiponectin levels, CRP and pro-inflammatory interleukines IL1, IL.6 and TNFalpha.

**Results:**

All parameters listed above except the cytokine were improved at D20, so that 4 subjects among 14 still presented the MS. After returning to home, these parameters remained stable.

**Conclusion:**

The efficacy of therapeutic lifestyle modifications with education and exercise and diet was demonstrated, but the compliance to the new healthy lifestyle initiated during the cure was not optimal.

## Introduction

Increase of body mass index (BMI) is a major risk factor of metabolic illness [1], in relation to abdominal obesity [2]. Visceral fat, as reflected by waist circumference, is linked to a lot of metabolic abnormalities like impaired glucose tolerance, dyslipidemia, high blood pressure (BP). This metabolic profile, called metabolic syndrome (MS), is associated with an increased risk of cardiovascular diseases (CVD) and type 2 diabetes prevalence [3]. In developed countries, its prevalence is increasing and is mainly linked to obesity and age [4].

The International Diabete Federation (IDF) has defined MS criteria as the co-occurrence of any three of the five following abnormalities: abdominal obesity (waist circumference > 94 cm in men and > 80 in women), dyslipidemia (triglyceridemia > 1,5 mmol/l, HDL cholesterol < 0.4 g/l in men and < 0.5 g/l in women), BP > 130/85 and/or treatment, and fasting glycemia > 5.55 mmol/l and/or treatment [5].

MS is acknowledged as a major cardiovascular risk factor of morbidity and mortality [6,7]. Moreover, it is associated with other metabolic abnormalities as a chronic systemic inflammation attested by an increase in blood IL-1, [8], IL-6, [9] and TNF-alpha [10] and correlated with visceral fat and insulinoresistance [11]. Physical-activity may increase systemic inflammation [12], although some studies showed a long-term reduction [13].

Some studies relate that MS may be reduced by change in diet habits, physical exercise and/or drug treatment [6]. Diet changes often consist in significant caloric intake reduction (at least 500 kcal/day). Physical-activity generally associates endurance (low intensity) and resistance (intensity higher than VO2max) exercises. However, the nutritional diets and/or the physical activities have been studied only for short term period (few weeks) and long-term effects remain unknown. A bad compliance, often observed in the subjects after acute treatment, seems responsible of treatment failure when patients return to routine life conditions.

In this survey, we analysed the specific effects on weight, glucido-lipidic metabolism and regulation, inflammation and adipokines, of an intervention program including two parts: a cure, or a three-week residential program during which they stayed in a medical establishment, with control on diet and physical-activity, and a six-month follow-up post-cure.

## Subjects and Methods

Fourteen volunteers presenting the symptoms of the metabolic syndrome, 10 men, 4 women, 62.9 ± 6.9 years, were enrolled by their general practitioner. For inclusion in the study, they underwent a clinical examination by the same physician of our group; briefly, we used the International Diabetes Federation (IDF) criteria defined in 2005 [5]. All of them should have no medications known to affect cholesterol measures, no medical affections contra-indicating the physical-activity and restricting diet in the past year. All participants completed this pilot study. This study was approved by the local ethical "Committee for the Protection of the Person for Research in Biology" (CPPRB).

### Study Design

**Figure 1:**
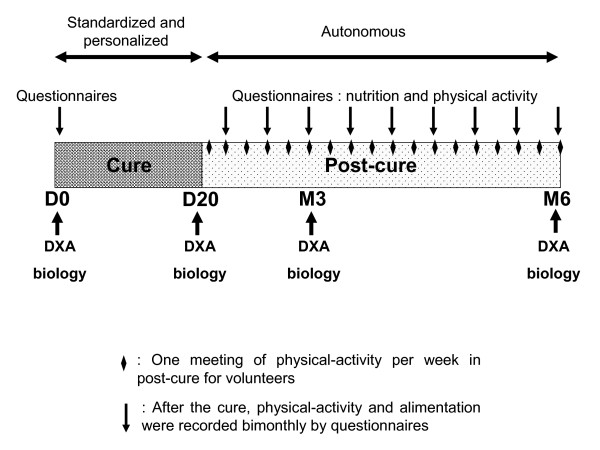
**Study design: a three-week intervention with standardized and personalized diet and physical activity, followed by an autonomous period for diet and physical activity accompanied by a weekly recall for physical activity **.

The study design is displayed in Figure 1. It lasted six months and included three chronological steps: before entering the cure (Day 0 : D0), the three-week cure (D0 to D20) and following the cure (D20 to Month 6 : M6). Clinical, biological and body composition parameters were assessed at D0, D20, M3 and M6.

At D0, basal metabolic rate (BMR) was calculated using the equations of Black [14]: (Basal Metabolic Rate = 0.963. weight^0.48^.height^0.50^.age ^0.13 ^for women, MB = 1.083. weight^0.48^.height^0.50^.age ^0.13 ^for men). Anthropometrical and clinical values (weight, size height, BMI, waist circumference, blood pressure), body composition by DXA and biological parameters were assessed. Before entering the cure, each patient completed questionnaires concerning his food intake and his physical-activity over a week. Daily Energy Intake (DEI) and daily energy expenditure (DEE) were estimated from the self reported questionnaires. The food intake questionnaires allowed us to detect possible deficiencies of the regime.

During the three-week cure, the patients carried out daily individually adapted physical activities with a coach: walking (2 h), aquagym, fitness (1 h); The exercise intensity was stated between 40 to 60% of the heart rate reserve (maximal theoretical heart rate - resting heart rate), using a heart rate recorder (Polar 4000). They followed this program six days a week. The 7th day they walked only. Their diet allowance was managed by a dietician. They also followed basic lectures regarding metabolic syndrome, nutrition physiology, cooking and physical-activity. The aim of the educational accompaniment was to teach them the lifestyle they need to self-manage to keep benefit of the cure after its end.

At D20, the patients came back to home and thus became autonomous, i.e. they have to manage the program by themselves. Thereafter, one meeting of physical-activity per week (fitness or aquagym) was organized in order to maintain the emulation and compliance to the program. Their physical-activity and their alimentation were recorded bimonthly by questionnaires. They could ask a dietician and a coach if they had any questions. Clinical and psychological follow-up was evaluated by a physician.

### Daily Energy Intake, physical activity index and balance

Estimation of DEI was based on questionnaires before and after the cure (three-day food record once every 15 days). Daily energy expenditure (DEE) was quantified by collecting time and intensity of each physical activity and the physical activity index (PAI = DEE/BMR) was calculated [15]. Each day during the cure, the patients received standardized and personalized balanced meals as established by dieticians. The aim was to rebalance macronutrients equilibrium as follows: 30 to 35% by lipids, 15 to 20% by proteins and the remaining by carbohydrates.

Daily physical activity was set for each subject in order to obtain a physical activity index equal to 1.4 [15,16]. Total daily food intake was calculated for each patient in order to reach a negative energy balance (EB = DEE-DEI) of 500 kcal at least vs usual intake.

### Biometry and Anthropometry

All subjects completed medical examinations. Body height was measured with a stadiometer, body mass was determined using an electronic scale and BMI was calculated as W/H^2 ^. Waist circumference and blood Pressure were recorded. Body composition was assessed by dual energy X-ray absorptiometry (DXA, Hologic QDR Delphi series; Waltham, USA). The in vivo coefficients of variation were 4.2 and 0.48% for fat and lean mass respectively. Central fat, (approximation of the visceral fat), was assessed with DXA. It consisted to measure the % fat in a rectangle defined from the upper edge of the second lumbar vertebra to the lower edge of the fourth lumbar vertebra. The vertical sides of this area were the continuation of the lateral sides of the rib cage [17]. All measures for a given parameter were done by the same investigator.

### Biochemical Measurements

Fasting blood samples were drawn between 7.30 and 8.30 a.m. on suitable tubes, aliquoted and stored at -80°C until analyses. Basic biology (glycaemia, blood lipids, creatinin, CRP, alpha 1-acid glycoprotein (α1GPA),) was assayed in the biochemistry laboratory of the University Hospital. Renal clearance was assessed following the Cockcroft formula [18]. All other biochemical determinations were made using commercial kits, following manufacturer recommendations, including sampling step, allowing the best performances of coefficient of variation (CV) and sensitivity.

Serum insulin was assayed by ELISA (Ins-ELISA, Bio-Line, Belgium; intra-inter assay CV of 3.0-5.3%, 4.5-9.5% and sensitivity of 4 ng/ml).

Pro-inflammatory cytokines (TNF-alpha, IL-1 and IL-6) were assayed by ELISA with commercial kits (AbCys SA, Paris, France).

Sensitivity, intra- and interassay CVs were respectively 3.83 pg/ml, 6.9% and 7.4%, for TNF-alpha, 1.6 pg/ml, 5.4% and 10% for IL-1, 0.92 pg/ml, 3.4% and 5.2% for IL-6.

The adipokines leptin and adiponectin were assayed using commercial kits (AbCys SA, Paris, France); sensitivity, intra- and interassay CVs were respectively, 0.17 ng/ml, 3% and 3.2% for leptine, 4.1 ng/ml, 4% and 5.5% for adiponectine. All components were assayed in the same batch in order to avoid inter-assay variations.

### Statistical Analysis

Gaussian distribution of the data was tested by the Kolmogorov-Smirnov test. Data are presented as mean ± Standard Error (SE). A Newman-Keuls post-hoc test for repeated measurements was used for normally distributed data and a nonparametric Wilcoxon test for non-normally distributed data. Relationships between balance energy, physical activity and others data were assessed either by Pearson correlation or by multiple regression analysis. Significance was accepted at the *p <*0.05 level. Statistical procedures were performed by using Statview (version 5.0; SAS institute Inc, Cary, North Carolina, USA).

## Results

### Descriptive characteristics of volunteers before the cure

The main parameters of MS are presented in Table 1; they revealed elevated lipidemia, glycaemia and BP. Usual food intake reported by questionnaires highlighted various food disorders. All patients had a high lipid (44,5% ± 4.6 of DEI) and a low carbohydrate consumption (39.2% ± 5.5 of DEI), with a too elevated high/low glycaemic index ratio (34.7 ± 10.9). Mean cholesterol consumption was 341 ± 97 mg/d and saturated fatty acid represented 16.6 ± 3.2% of DEI (39.5% of the lipid intakes). The mean DEE before the cure was 1983 ± 228 kcal/day corresponding to a low PAI of 1,22 ± 0.09.

**Table 1:** Descriptive characteristics of the subjects at baseline: MS markers, daily energy expenditure, physical activity index and food intakes.

	Mean ± SE	Minimum	Maximum
Age (years)	62,9 ± 6,9	52	79
Weight (kg)	85,6 ± 10,3	62,8	106,6
MS parameters			
BMI (kg/m^2^)	30,4 ± 3,8	24,3	36,9
Waist circumference (cm)	105,07 ± 7,39	93	119
Blood Pressure (mmHg)	137/83 ± 11/5	120/80	155/95
Triglycerides (mmol/l)	1,68 ± 1,15	0,9	5,1
HDL (mmol/l)	1,44 ± 0,42	0,86	2,3
Glycaemia (mmol/l)	6,15 ± 1,86	4,5	11
Basal Metabolic Rate	1625,9 ± 177,8	1171,1	1927,2
Physical Activity Index	1,22 ± 0,09	1,06	1,38
Daily Energy Expenditure (kcal/d)	1983,3 ± 228,7	1347,2	2277,2
Daily Energy Intake (kcal/d)	2072,8 ± 556,3	1562,0	2742,7
Daily Energy Intake (kcal/kg/d)	23,8 ± 6,5	16,9	31,9
Percentage of each macronutriments in the food			
% Carbohydrates	39,19 ± 5,55	34,98	47,06
of which % Fast carboydrates	13,85 ± 5,32	5,37	23,84
% Lipids	44,53 ± 4,66	37,86	53,97
% Proteins	16,31 ± 1,5	14,05	19,18
Protein intake (g/kg/d)	0,91 ± 0,26	0,63	1,23

### Descriptive characteristics during the cure

#### Intervention

Evolution of food intake is presented in Table 2. Evolution of energy balance and PAI are presented in Table 3. DEI decreased and DEE increased during the cure, both significantly, resulting in a negative balance. The physical activity index was set at 1.42 ± 0.07. The distribution in macronutrients decreased for lipids and increased for carbohydrates and proteins. The saturated fatty acids were reduced as well as cholesterol.

**Table 2:** Changes in food intakes: Percentage of macro-nutriments including saturated fatty acid, food cholesterol and protein contribution are given

	D0	D20	M3	M6	p-value
% Carbohydrates	39,2 ± 5,5	48,3 ± 2,5	42,5 ± 4,6	39,1 ± 3,4	**D0 vs D20, M3 : p < 0.005**
% Lipids	44,5 ± 4,7	32,8 ± 2,1	38,2 ± 4,7	41,7 ± 3,6	**D0 vs D20 : p < 0,0001**
% Satured Fatty Acid	16,6 ± 3,2	12,9 ± 1,4	14,9 ± 1,4	15,8 ± 1,8	**D0 vs D20, M3, M6 : p < 0.0001** **D20 vs M3, M6 : p < 0,0001**
Food cholesterol (mg/d)	341 ± 97	271 ± 30	339 ± 160	357 ± 57	**D0 vs D20 : p < 0,05**
% Proteins	16,3 ± 1,5	18,9 ± 0,8	19,4 ± 0,19	19,4 ± 1,3	**D0 vs M3, M6 : p < 0,05** **D20 vs M3, M6 : p < 0,05**
Protein Intake (g/kg/d)	0,91 ± 0,26	0,85 ± 0,11	0,94 ± 0,19	0,96 ± 0,17	**D0 vs M3, M6 : p < 0,05** **D20 vs M3, M6 : p < 0,05**

**Table 3:** Changes in energy balance, physical activity index, MS parameters and body composition during cure and follow-up.

	D0	D20	M3	M6	p-value
Energy Balance (kcal/d)	92 ± 521	-751 ± 147	-521 ± 304	-413 ± 304	**D0 vs D20, M3 : p < 0.005** **D20 vs M3, M6 : p < 0,02**
Physical Activity	1,22 ± 0,09	1,42 ± 0,07	1,31 ± 1,97	1,30 ± 0,07	**D0 vs D20, M3, M6 : p < 0,035** **D20 vs M3, M6 : p < 0,003**

Metabolic Syndrome parameters :					
BMI (kg/m^2^)	30,4 ± 3,8	29,6 ± 3,6	28,4 ± 2,9	28,6 ± 2,9	**D0 vs D20, M3, M6 : p < 0.0001** **D20 vs M3, M6 : p < 0,0001**
Waist circumference (cm)	105,07 ± 7,39	102,68 ± 7,86	99,15 ± 6,31	98,92 ± 7,44	**D0 vs D20, M3, M6 : p < 0,05** **D20 vs M6 : p < 0,05**
Blood Pressure (mmHg)	137/83 ± 11/5	130/80 ± 13/5	129/80 ± 15/6	132/78 ± 16/9	**Systolic BP D0 vs D20, M3 : p < 0,05 **; Diastolic BP : NS
Triglycerides (mmol/l)	1,68 ± 1,15	1,19 ± 0,34	1,17 ± 0,40	1,27 ± 0,61	NS (p = 0,17)
HDL (mmol/l)	1,44 ± 0,42	1,49 ± 0,31	1,31 ± 0,31	1,58 ± 0,46	**D20 vs M3 : p = 0,005** **M3 vs M6 : p = 0,005**
Glycaemia (mmol/l)	6,15 ± 1,86	6,26 ± 1,76	5,7 ± 1,97	6,28 ± 2,34	**D20 vs M3 : p = 0,0409** **M3 vs M6 : p = 0,0121**

Body composition measured by DEXA :					
Weight (kg)	85,59 ± 10,31	83,31 ± 9,90	80,08 ± 9,53	80,73 ± 10,38	**D0 vs D20, M3, M6 : p < 0,0001** **D20 vs M3, M6 : p < 0,0001**
Lean (g)	58287 ± 7473	57323 ± 7561	56488 ± 7892	56798 ± 7991	**D0 vs D20, M3, M6 : p < 0,0001**
Total fat (g)	29542 ± 9752	26737 ± 9377	25524 ± 8939	26213 ± 9580	**D0 vs D20, M3, M6 : p < 0,0001**
Visceral fat (g)	3277 ± 1235	2839 ± 1216	2695 ± 1069	2527 ± 1005	**D0 vs D20, M3, M6 : p < 0,0001** **D20 vs M6 : p < 0,0001**
Bone Mineral Density (g)	2421 ± 381	2447 ± 391	2416 ± 379	2456 ± 402	**D0 vs M6 : p = 0,007** **M3 vs M6 : p = 0,007**
Fat percentage (%)	32,33 ± 7,92	30,51 ± 8,26	29,9 ± 8,15	30,24 ± 8,33	**D0 vs D20, M3, M6 : p < 0,0001**

#### Effects (Table 3 and 4)

The association of diet and physical-activity reduced significantly the body weight, BMI, waist circumference and systolic BP. Fifty seven percent of weight loss accounted for fat mass. Among fat mass, central fat decreased significantly. The rates of HDL remained stable and triglyceridemia showed a trend to decrease (NS). Other blood lipid parameters decreased significantly. The high insulinemia and HOMA-IR noted before the cure were normalized at D20. Creatininemia and creatinin clearance remained stable. Inflammatory parameters didn't change significantly.

**Table 4:** Evolution of blood markers of glucid and lipid metabolisms, pro-inflammatory factors and adipokines during cure and follow-up.

	D0	D20	M3	M6	p-value
Glucidic parameters :					
Insulinemia (microU/ml)	20,96 ± 24,13	5,73 ± 0,53	5,86 ± 0,68	6,15 ± 0,51	**D0 vs D20, M3, M6 : p < 0,01** **D20 vs M6 : p = 0,028**
Glycaemia (mmol/l)	6,15 ± 1,86	6,26 ± 1,76	5,7 ± 1,97	6,28 ± 2,34	**D20 vs M3 : p = 0,0409** **M3 vs M6 : p = 0,0121**
HOMA-IR	6,71 ± 8,86	1,62 ± 0,62	1,48 ± 0,52	1,73 ± 0,75	**D0 vs D20, M3, M6 : p < 0,01**

Other lipidic parameters :					
Total cholesterol (mmol/l)	6,08 ± 1,46	5,07 ± 0,95	5,13 ± 1,25	5,56 ± 0,89	**D0 vs D20, M3 : p = 0,001**
LDL (mmol/l)	3,79 ± 1,21	3,05 ± 0,87	3,47 ± 1,25	3,43 ± 0,95	**D0 vs D20 : p = 0,003**
Cholesterol total/HDL	4,65 ± 2,18	3,51 ± 0,85	4,10 ± 1,24	3,83 ± 1,37	**D0 vs D20. M6 : p = 0,011**
LDL/HDL	2,89 ± 1,31	2,15 ± 0,75	2,82 ± 1,21	2,42 ± 1,11	**D0 vs D20, M6 : p = 0,001** **D20 vs M3 : p = 0,001** **M3 vs M6 : p = 0,001**

Pro-inflammatory factors :					
CRP (mg/l)	3,92 ± 2,40	3,83 ± 3,78	3,04 ± 3,23	2,45 ± 1,62	**D0 vs M6 : p = 0,0303**
α1GPA (g/l)	0,84 ± 0,16	0,79 ± 0,20	0,75 ± 0,15	0,81 ± 0,16	NS (p = 0,18)
Pro-inflammatory cytokines :					
IL1 (picog/ml)	2,96 ± 0,15	2,95 ± 0,16	2,87 ± 0,04	2,93 ± 0,12	NS (p = 0,33)
IL6 (picog/ml)	1,17 ± 0,34	2,95 ± 0,16	2,87 ± 0,04	2,93 ± 0,12	NS (p = 0,36)
TNFα (picog/ml)	11,06 ± 0,42	10,99 ± 0,67	10,78 ± 0,70	10,75 ± 0,51	**D0 vs M6 : p = 0,0355**

Adipokines :					
Leptin (nanog/ml)	14,34 ± 7,73	9,57 ± 6,27	9,78 ± 8,59	11,00 ± 10,34	NS (p = 0,1)
Adiponectin (μg/ml)	12,98 ± 4,82	15,56 ± 2,28	14,75 ± 4,80	15,45 ± 3,87	NS (p = 0,13)

### Descriptive characteristics following the cure

#### Lifestyle (Table 2 and 3)

Dietetic recommendations were given every 15 days to keep a negative energy balance. The protein contribution increased up to 0,96 ± 0.17 g/kg/d. A progressive increase in DEI was reported jointly to a reduction in DEE; the food intake reduction remained significant until M3 concerning lipids and glucids; this reduction was not significant at M6. The patients gradually came back to their former dietary habits with an increasing lipid intake, a reduction of carbohydrates contribution with an increasing high/low glycaemic index ratio.

#### Effects (Table 3, 4)

The weight loss and the central fat loss continued (D20 vs. M6 : p < 0.0001), as well as the reduction of BMI and waist circumference. The insulinemia and HOMA-IR remained at the normal value of D20. The pro-inflammatory marker CRP decreased significantly whereas the inflammatory cytokines IL1, IL6 and TNF alpha didn't change.

### Descriptive assessment at six months

At M6, we recorded the following changes vs D0 : a weight loss of 4.36 kg (p < 0.0001), a reduction of BMI of 1.6 kg/m2 (p < 0.0001), a fall of systolic blood pressure of 4.46 mmHg (NS) and 5.39 mmHg for diastolic blood pressure (NS), a waist circumference reduction of 5.08 cm (p < 0.05), and a reduction of triglycerides of 0.364 mmol/l or 22.7% (NS), an increase in the HDL-cholesterol of 0.144 mmol/l or 10.14% (NS). The total cholesterol/HDL and LDL/HDL ratio remained statistically decreased at M6. The insulinemia and HOMA-IR normalised quickly (D20) and remained stable thereafter. CRP and TNF-alpha decreased significantly. The pro-inflamatory factors (CRP, Alpha1GPA) decreased significantly from D0; no changes were recorded for the inflammatory cytokines.

No significant changes were recorded for the adipokines leptine and adiponectine;

## Discussion

The main findings of this longitudinal study were that a restrictive diet associated to physical-activity induced a decrease in cardiovascular risk factors linked to MS. All the individuals enroled responded to the criteria of the metabolic syndrome as defined by IDF [5]. At M6, 4 patients among 14 still presented the characteristics of MS, which means that 71% of the group had observed positive results, and thus a good compliance to the program, probably in relation with the 3 weeks residential program which acted as a booster.

We didn't split the results between genders, since there were 4 females only, all in post menopausal stage. There was no control group in account of ethical limitations. As highlighted by the questionnaires, MS was mainly due to bad dietary habits, particularly for the quality and to a very low level of physical activity (Table 1 and 2). The challenge of this intervention was to reverse their bad glucido-lipidic blood constants by modifying their lifestyle only, without any medication.

The mediterranean diet has been shown to exert a favourable benefit/hazard ratio to reduce the cardiovascular morbi-mortality associated with MS [19]. Adding endurance exercise training to a dietary weight-reduction program further improves parameters of MS and body composition [20-22].

The reduction in the prevalence of MS obtained using this natural method in our study is similar, even better, to those using medications like rimonabant [23] (anorexigenic) or methformin [24] (antihyperglycaemic), in agreement with the conclusion of the "Diabetes Prevention Program" [24] without risks of adverse effects, particularly psychic disturbancies [25].

Globally, all the parameters of the MS were improved: weight, waist circumference, total and central fat weight were diminished at D20 and remained stable till M6 (Table 3). Systolic blood pressure followed the same evolution.

Although we calculated a caloric deficit of 458 kcal/d at M6, the results recorded in Table 3 show that body weight remained stable from M3 to M6; This may be due to an under-statement of DEI by the patients together with an over-statement of DEE, but another explanation is possible: it was shown by Apfelbaum that a reduction of the energy intake with a balanced protein intake reduced the BMR by 20%, while the active tissue mass remained constant [26]. Such a 20% reduction in BMR will account for 325 kcal decrease of daily allowance in our group. Consequently, a reduction in DEI taking into account a recalculation of BMR would have been useful.

Glucidic metabolism was significantly improved by the cure. At D0, the patient were not hyperglycaemic but showed a tendency to insulin insensitivity (high insulinemia) which was reversed at D20 and remained stable till M3 (Table 4); however insulinemia showed a slight tendency to rise between M3 and M6.

This index of the MS was then significantly improved by the intervention as previously reported in young people [27].

Blood triglycerides, total and LDL-cholesterol decreased at D20 and didn't vary after; conversely, HDL Chol was unchanged at D20 but showed an increase between M3 and M6; this improvement must probably be considered in account of the long term increase of the physical activity index (Table 3) [28].

The adiopokines which levels are generally correlated, positively for leptin and negatively for adiponectin with the fat mass [27], didn't change significantly, although a significant reduction of fat mass in the subjects of this study.

Parameters of MS were improved without changes in chronic systemic inflammation (Table 4), as was reported in other studies [29,30]. In fact, basal values at D0, even high, were not out of the normal limits for these markers. The pro-inflammatory factor CRP only showed an improvement from D0 to M3 and M6, corresponding to a cardiovascular risk [31] and MS [32] decreases.

## Conclusion

Globally, the efficacy of therapeutic lifestyle modifications with education and counselling to exercise and diet was demonstrated, as previously reported [33]. In our study, the three weeks residential program allowed a rapid improvement in parameters of the MS, since 4 subjects only among 14 still presented the MS; after returning home, although advices have been given along the 6 months follow-up period, the MS criterions didn't continue to progress, they remained stable only.

This is an important issue because only long-term changes in lifestyle can reduce morbi-mortality of MS. In the literature, several behavioural techniques have been used to improve patients' adherence [34]. Future studies should investigate the way of improving long-term compliance in the treatment of MS, in particular the best duration of a residential program on a larger population for allowing new lifestyle habits.

## Competing interests

The authors declare that they have no competing interests.

## Authors' contributions

FD has participated as PhD student and main investigator. BL, DC and GL contributed to the conception of the protocol, data analysis and manuscript drafting.

RC and ED had responsibilities in daily diet and physical activity management. GL has revised and given final approval of the manuscript. All authors read and approved the submitted manuscript.
